# Empowering Health Care Workers on Social Media to Bolster Trust in Science and Vaccination During the Pandemic: Making IMPACT Using a Place-Based Approach

**DOI:** 10.2196/38949

**Published:** 2022-10-17

**Authors:** Shikha Jain, Serena R Dhaon, Shivani Majmudar, Laura J Zimmermann, Lisa Mordell, Garth Walker, Amisha Wallia, Halleh Akbarnia, Ali Khan, Eve Bloomgarden, Vineet M Arora

**Affiliations:** 1 Department of Medicine University of Illinois at Chicago Chicago, IL United States; 2 Chicago College of Osteopathic Medicine Midwestern University Downers Grove, IL United States; 3 College of Medicine University of Illinois at Chicago Chicago, IL United States; 4 Department of Preventive Medicine, Department of Internal Medicine Rush University Chicago, IL United States; 5 Loyola University Stritch School of Medicine Maywood, IL United States; 6 Department of Emergency Medicine Northwestern University Evanston, IL United States; 7 Department of Endocrinology, Metabolism and Molecular Medicine Department of Medicine Northwestern University Evanston, IL United States; 8 Advocate Condell Medical Center Libertyville, IL United States; 9 Oak Street Health Chicago, IL United States; 10 NorthShore University HealthSystem Evanston, IL United States; 11 University of Chicago Pritzker School of Medicine Chicago, IL United States

**Keywords:** misinformation, COVID-19, place-based, infodemic, infographic, social media, advocacy, infodemiology, vaccination, health care worker, policy maker, health policy, community health

## Abstract

**Background:**

Given the widespread and concerted efforts to propagate health misinformation on social media, particularly centered around vaccination during the pandemic, many groups of clinicians and scientists were organized on social media to tackle misinformation and promote vaccination, using a national or international lens. Although documenting the impact of such social media efforts, particularly at the community level, can be challenging, a more hyperlocal or “place-based approach” for social media campaigns could be effective in tackling misinformation and improving public health outcomes at a community level.

**Objective:**

We aimed to describe and document the effectiveness of a place-based strategy for a coordinated group of Chicago health care workers on social media to tackle misinformation and improve vaccination rates in the communities they serve.

**Methods:**

The Illinois Medical Professionals Action Collaborative Team (IMPACT) was founded in March 2020 in response to the COVID-19 pandemic, with representatives from major academic teaching hospitals in Chicago (eg, University of Chicago, Northwestern University, University of Illinois, and Rush University) and community-based organizations. Through crowdsourcing on multiple social media platforms (eg, Facebook, Twitter, and Instagram) with a place-based approach, IMPACT engaged grassroots networks of thousands of Illinois health care workers and the public to identify gaps, needs, and viewpoints to improve local health care delivery during the pandemic.

**Results:**

To address vaccine misinformation, IMPACT created 8 “myth debunking” infographics and a “vaccine information series” of 14 infographics that have generated >340,000 impressions and informed the development of vaccine education for the Chicago Public Libraries. IMPACT delivered 13 policy letters focusing on different topics, such as health care worker personal protective equipment, universal masking, and vaccination, with >4000 health care workers signatures collected through social media and delivered to policy makers; it published over 50 op-eds on COVID-19 topics in high-impact news outlets and contributed to >200 local and national news features. Using the crowdsourcing approach on IMPACT social media channels, IMPACT mobilized health care and lay volunteers to staff >400 vaccine events for >120,000 individuals, many in Chicago’s hardest-hit neighborhoods. The group’s recommendations have influenced public health awareness campaigns and initiatives, as well as research, advocacy, and policy recommendations, and they have been recognized with local and national awards.

**Conclusions:**

A coordinated group of health care workers on social media, using a hyperlocal place-based approach, can not only work together to address misinformation but also collaborate to boost vaccination rates in their surrounding communities.

## Introduction

With the COVID-19 pandemic came an onslaught of misinformation that undermined the ability to keep the pandemic in check. Because misinformation has been declared a public health crisis by the surgeon general of the United States, individual clinicians are advised to partake in tackling misinformation, particularly when using social media [1]. However, addressing health misinformation can be particularly challenging for individual health care workers who were often serving on the frontlines in the pandemic. Engaging in such activities is even more challenging for health care workers with caregiving responsibilities or those whose communities were disproportionately affected by the pandemic [2].

Health care professionals continue to be trusted sources of information, though fewer Americans trust social media as a source of information [3]. Studies show that social media posts from clinicians are more trusted than posts by political figures [4]. However, health care workers often face attacks and harassment for spreading medical information during the pandemic, particularly due to the increasing polarization of our populace on political lines, with respect to mitigation strategies [5,6].

Although social media has the ability to bring together health care workers to tackle misinformation and promote public health on a national scale in the digital space, there are still major questions that remain unanswered about such efforts. For example, linking social media efforts to actual patient or clinical outcomes continues to be a major challenge, especially on social media, where clinicians are often distributed over large distances.

A specific place-based approach, used across other sectors focused on both the social and physical environment of a community, may be particularly impactful to address misinformation, while simultaneously engaging a local community to improve outcomes [7,8]. During a pandemic, this may be of particular importance, for example, for vaccination rates in a community. To date, few social media efforts describe such a place-based approach to address misinformation, subsequently affecting health processes or outcomes in public health, such as vaccination rates in a community. The aim of this paper is to provide a descriptive process analysis of a multimodal initiative that specifically coupled place-based theory with a social media strategy to combat misinformation related to COVID-19. The goal was to mobilize a virtual health care community to reduce COVID-19 vaccine hesitancy and improve vaccine access in underserved areas served by this community.

## Methods

The Illinois Medical Professionals Action Collaborative Team (IMPACT) is an interdisciplinary coalition of health care professionals, founded in March 2020 and established as a 501(c)(3) nonprofit organization in 2022, in response to the COVID-19 pandemic. IMPACT [9] leverages social media and novel partnerships to (1) identify and amplify public health needs and inequities in care delivery, (2) address needs and gaps by rapidly disseminating evidence-based information, (3) connect groups to resources, and (4) advocate for science-based policy.

IMPACT started as an extension of a well-established, closed clinician social media–based group (on Facebook) of >2800 verified physician members in the Chicagoland area. The Facebook group was created in 2015 and is moderated by an IMPACT cofounder and one of the authors (LZ). In March 2020, a post by a clinician in this Facebook group highlighted public health concerns regarding the community spread of COVID-19 and brought attention to the need for preventive measures (eg, social distancing). At the same time, Chicago-based social media physician advocates (VA, SJ, and AK) were sharing similar concerns on another social media platform (Twitter), sharing images of densely packed crowds at both Chicago Saint Patrick’s Day celebrations and Chicago’s O’Hare International airport [10-12]. As a result, members of the group, now known as IMPACT, mobilized on social media to write a letter that amassed over 300 signatures from verified health care workers (crowdsourced on social media and shared networks) and delivered it to the governor to address the concerns of the health care community [13,14]. The concerns stemmed primarily from lack of social distancing observed in local communities, despite the continued reinforcement by IMPACT members’ own health care facilities of the need to distance in the hospitals to avoid being infected. The rapid response strategy of crowdsourcing on Illinois-based health care worker networks on Facebook, followed by an amplification on Twitter by Chicago-based social media health care advocates and leaders, demonstrated that a coordinated place-based (eg, a local coordinated community) strategy could be effective on social media to influence policy changes and help advise the community during this challenging time. To link these various efforts across social media platforms, IMPACT was formed by team members across disciplines and expertise with social media advocacy work across multiple platforms. Members of the organization were from diverse backgrounds, including community members and organizations, health care workers, and health care organizations. Networks and expertise were combined to create an infrastructure to (1) identify community needs (ie, public health measures), (2) garner information about resource allocation and misallocation, (3) rapidly inform and amplify important trusted information, and (4) alert policy makers and the community of key trusted information or areas of confusion. Later, with the help of trainees (ie, students and residents) and other volunteers, IMPACT expanded the place-based approach and social media strategy to other social media outlets such as Instagram, LinkedIn, and TikTok (@rubin_allergy).

IMPACT immediately recognized that this place-based social media strategy was effective in communicating and amplifying issues that community-based organizations were facing, including personal protective equipment (PPE) procurement and the need for masks. IMPACT subsequently partnered with 2 student-led, community-based organizations—MasksNow Illinois and Get Us PPE Chicago—to help support and fundraise for masking in disadvantaged communities, homeless shelters, and nursing homes. For example, when a need for cloth masks was identified in Little Village—a predominantly Spanish-speaking community in Chicago’s West Side—IMPACT leaders connected community leaders with MasksNow Illinois to supply over 15,000 masks to this community. As a result of this early advocacy, a feature story on IMPACT appeared in the Chicago Tribune that focused on the origins of the group, a derivation primarily from physician mothers through the Facebook Chicago group [15]. This piece led to a weekly segment for one of our founders (SJ) during the morning news on Fox32—the local Fox television affiliate. The recurring news segment served as a vehicle to provide information to the community about public health measures [16].

As the COVID-19 pandemic progressed, it was abundantly clear that local communities would benefit greatly from the scientific advancement through COVID-19 vaccines. Unfortunately, it was also recognized that progress would be impeded through inequities in both distribution and uptake in vaccination rates at the local level. IMPACT, therefore, leveraged its social media platforms and strategy to specifically begin to communicate crucial trusted information about vaccines in addition to helping support vaccination events across the Chicagoland area.

IMPACT created infographics to rapidly and effectively communicate key messages to other health professionals and to the general public [17]. Infographics were created by a core “digital media” team of volunteer student interns, a physician lead, and IMPACT cofounders. Infographics drafted by this core team were then reviewed and edited for a lay audience with an eighth-grade literacy. Citations and dates were included and confirmed in each infographic. Infographics were then reviewed by the broader IMPACT team and translated into Spanish by bilingual IMPACT members. Once approved, infographics were shared across social media. As of April 2022, IMPACT designed and shared over 60 infographics.

In addition to social media and traditional media, IMPACT was also called upon to provide grassroots educational efforts with trusted messengers to reach out to the communities most impacted by COVID-19. Since the beginning of the pandemic, over 80 virtual community town halls have taken place in both English and Spanish to inform communities about COVID-19. In addition to these community town halls, IMPACT was contacted by a National Library of Medicine All of Us grantee to generate English and Spanish “train the trainer” videos and training for the Chicago Public Library librarians to serve as trusted messengers to the community on the COVID-19 vaccine topic.

IMPACT additionally expanded and cosponsored large vaccination efforts across Chicago and the greater Chicagoland areas. Many of the vaccination events have occurred in areas of low vaccination status and in partnership with community organizations, places of faith, and schools already trusted by local communities. When Emergency Use Authorization of COVID-19 vaccines for children aged 5-11 years was announced, IMPACT addressed concerns of the communities, and we continue to collaborate with partners to vaccinate and protect children.

The key to the IMPACT strategy has been multistakeholder collaborations. IMPACT additionally partnered with multiple academic institutions in the Chicagoland area to launch key educational initiatives. At the Pritzker School of Medicine at the University of Chicago, it developed and launched a new course on misinformation and science communication for early-year medical students; one of the students’ final projects was featured [18] at a national symposium sponsored by the Association of American Medical Colleges [19]. Students at the University of Chicago and the University of Illinois also participated in an op-ed accelerator, which has resulted in over 12 op-eds with student first authors. At the University of Illinois, IMPACT formed a formal internship program with medical students involved in the urban medicine elective to train the next generation of physicians in advocacy.

IMPACT continues to support and develop vaccination events with community partners, create information campaigns across the digital and traditional media space, and pioneer community events with trusted messengers to educate the public and address misinformation surrounding the ongoing pandemic. All of IMPACT’s work rests on the volunteer time of health care workers or health professional trainees who do this work in addition to their daytime work and responsibilities.

## Results

Our evaluation centers around IMPACT’s activities in public policy and the media, social media campaign reach, as well as vaccine education and outreach in the Chicago community. Due to the nature of this work, we focus on the reach of our social media efforts, using a variety of metrics readily available on social media platforms, as well as both the reach and effectiveness of our social media vaccination efforts.

### Policy and Media Reach

IMPACT has delivered 13 policy letters with >4000 health care workers signatures collected through social media and delivered to policy makers and has published >50 op-eds in local and national media (eg, US News, Chicago Tribune, Health Affairs, USA Today, CNN, and Newsweek). IMPACT members have been featured in national media, including the New York Times, Time Magazine, Washington Post, Good Morning America, and National Public Radio and have appeared in >200 local and national news media, educating the public about COVID-19 mitigation and prevention.

### Social Media Reach

IMPACT social media campaigns have resulted in Facebook, Twitter, Instagram, and LinkedIn pages with >4000 followers, and posts earning >20,000 views on Facebook alone, as well as a newly verified Twitter account indicated by a blue checkmark. Successful campaigns included a social distancing hashtag (#6ftApartNotUnder) with >4000 tweets and millions of impressions, a universal mask mandate petition on change.org website with >113,000 signatures, a virtual #WhiteCoatsforBlackLives march with >1 million impressions, and COVID-19 data infographics with >400,000 impressions. The number of social media impressions is defined as the number of times content is displayed on a user’s screen or within their feed, regardless of whether it is clicked on or interacted with.

### Vaccine Education and Outreach

IMPACT created a “myth debunking” series of 8 infographics and a “vaccine information series” of 14 infographics that were shared across social media platforms and used by local schools, health departments, advocacy organizations, and community outreach events [20]. These two infographics series alone generated >330,000 impressions from social media based on an analysis of IMPACT social media metrics. Later, volunteers at community vaccine events were trained to use IMPACT infographics in printed forms as educational resources. Digital and print copies were also provided to community members to help inform them of the importance of vaccination.

To help improve vaccination rates, disparities in vaccination needs were rapidly identified through multiple sources (eg, Twitter, Chicago Facebook health care worker groups, emails, and messages to IMPACT) for health care workers not affiliated with health systems. An IMPACT clearinghouse for vaccine information (eg, registration and interest surveys) was created, procuring information rapidly through social media and professional networks [21]. In response to the concerns IMPACT raised regarding the accessibility of vaccines to those unaffiliated with hospitals, local health departments encouraged all health care entities to vaccinate non–system-affiliated health care workers. This work was highlighted in the Chicago Mayor’s weekly press conference with an IMPACT representative [22].

During the later phases of vaccination, when vaccines were available to the general public, IMPACT played a leading role, using social media networks to mobilize >700 health care workers along with >1000 nonmedical volunteers to staff vaccine events in multiple communities [21]. To date, IMPACT has organized or assisted in approximately 400 vaccine events, which have resulted in the vaccination of over 120,000 individuals in the Chicago region. This includes the administration of 5545 pediatric vaccine doses and 6456 booster doses given at 45 different pediatric and booster vaccination events since November 2021.

For this work, IMPACT has received multiple recognitions. The 2021 Community Activism Award from the Democrats of Northfield Township was presented to the group by Governor Pritzker. A community award, the Leadership Legacy Award, was granted by one of the first schools that had approached IMPACT for help. This school benefited from IMPACT’s virtual town halls for parents, students, and teachers, followed by actual on-site vaccine events, bringing the percentage of teachers vaccinated from under 50% to 95%. IMPACT was also one of the four recipients of the 2022 Innovations to Bolster Community Trust and Engagement in Science Award from the Association of American Medical Colleges [23]. One of our members (HA) also received the WGN News Remarkable Women Award for her efforts in organizing the vaccine clearinghouse and mobilizing volunteers to vaccinate Chicagoland communities [24].

## Discussion

This is the first description of a group of health care professionals using a predominantly web-based strategy on social media to engage in place-based advocacy during the COVID-19 pandemic. This paper reports on the relatively underreported area of health advocacy through social media, an approach with increasing relevance due to an increase in misinformation on social media and the need for health care professionals to address the needs of communities in real-time crises such as the COVID-19 pandemic. Social media crowdsourcing and digital collaborations with local stakeholders and various experts allowed the group to identify and counteract misinformation, identify and amplify the different needs of local communities, and direct both information and resources more equitably.

It is worth considering what is novel about this approach and when and why it might be successful. First, because the COVID-19 pandemic magnified health care disparities, it accelerated the need to develop innovative strategies to provide both information and resources to underserved communities on social media and on the ground. Although the creation of infographics to address misinformation is not new, repurposing infographics created primarily for use in the digital space to be used in physical forms at vaccination events can help “train the trainer” and highlight the role of community members as vehicles to dispel misinformation [25]. The pandemic also laid bare the vulnerabilities of the health care system and the interdependence of hospital capacity, public health messaging, and politics. Health care professionals are trusted voices in the community with the expertise to counter misinformation and advocate for the needs of their patients. Although individual efforts remain important and have been well described in health care in the past [26], this novel method of collective advocacy with a community or place-based approach has the ability to amplify and leverage evidence-based information on social media and other platforms, with the possibility of timely change for the public in real time. The use of a place-based approach is particularly unique in that, and if used as described, it can help leverage the power of social media engagement and mobilize the local community to fill gaps and address inequities identified at the community level. A place-based approach also lends itself to advocacy efforts, given that much of local policy is settled on a local and state level, particularly during the pandemic, as states and cities faced different community infection rates and differences in community needs. Therefore, such types of advocacy can be used to improve resource alignment and outreach in communities most in need.

There are limitations to this descriptive process analysis. The most significant limitation is the inability to measure the effectiveness of our social media interventions by measuring outcomes among social media users and community members exposed to our work; this requires formal research, and future research may include a randomized controlled trial as well as a qualitative study to further assess the effectiveness of our interventions. Outcomes demonstrating effectiveness include respondent knowledge, attitudes, perceptions, and practices, following exposure to our interventions. Future work may also include testing a more formal process of cocreation of infographics and other materials with members of audience communities. Nonetheless, there is value in the metrics of social media reach and the anecdotal evidence provided; this paper demonstrates a proof of concept and the feasibility of a place-based approach to health care advocacy through social media, laying the groundwork for formal research studies. There are also limitations to IMPACT’s entirely volunteer-based model described in this study; although IMPACT is now a 501(c)(3) nonprofit organization, we were initially unable to raise money and relied on the volunteer time of busy clinicians and students to execute this work. It is possible that a model with fundraising would have greater reach and effectiveness compared to our model.

In conclusion, IMPACT describes how a place-based approach on social media across multiple platforms can be repurposed not only to combat misinformation at the community level but also to advocate for science-based policies, engage stakeholders, and help direct resources to organizations and communities most in need. This proof-of-concept application of a social media strategy with a place-based approach may be useful to address other public health needs such as gun violence or the opioid epidemic. Further exploration of such approaches is warranted.

## Abbreviations

IMPACT: Illinois Medical Professionals Action Collaborative Team
PPE: personal protective equipment
